# Extended-spectrum beta-lactamase-producing *Enterobacteriaceae* related urinary tract infection in adult cancer patients: a multicenter retrospective study, 2015–2019

**DOI:** 10.1186/s12879-023-08023-3

**Published:** 2023-03-06

**Authors:** Guojing Wang, Yu Zhu, Shana Feng, Baojun Wei, Yujuan Zhang, Jingzhi Wang, Shengkai Huang, Shengling Qin, Xuan Liu, Bing Chen, Wei Cui

**Affiliations:** 1 Department of Clinical Laboratory, National Cancer Center/National Clinical Research Center for Cancer/Cancer Hospital, Chinese Academy of Medical Sciences and Peking Union Medical College, Beijing, 100021 China; 2 Department of Comprehensive Oncology, National Cancer Center/National Clinical Research Center for Cancer/Cancer Hospital, Chinese Academy of Medical Sciences and Peking Union Medical College, Beijing, 100021 China; 3 Department of Clinical Laboratory, Beijing Chaoyang Sanhuan Cancer Hospital, Beijing, 100023 China; 4 Department of Clinical Laboratory, Cancer Hospital of Huanxing Chaoyang District Beijing, Beijing, 100005 China

**Keywords:** Extended-spectrum beta-lactamase, Urinary tract infection, Cancer patients, Adult, Risk factors

## Abstract

**Background:**

The aim of this study was to investigate the prevalence and risk factors of extended-spectrum beta-lactamase (ESBL)-producing *Enterobacteriaceae* related urinary tract infections (UTI) in adult cancer patients.

**Methods:**

We conducted a retrospective study of three cancer hospitals centered on Cancer Hospital of Chinese Academy of Medical Sciences from 2015 to 2019. The clinical characters, risk factors and antimicrobial susceptibility of ESBL-producing *Enterobacteriaceae* UTI in adult cancer patients were described and analyzed.

**Results:**

A total of 4967 specimens of UTI were evaluated, of which 909 were positive. After excluding multiple infection bacteria, non-conforming strains, inconsistent pathological information, no drug sensitivity test or medical records, 358 episodes remained. Among them, 160 episodes belonged to ESBL-producing *Enterobacteriaceae,* while 198 were classified into non-ESBL group. The prevalence of ESBL UTI circled around 39.73 to 53.03% for 5 years. Subgroup analysis by tumor type revealed that 62.5% of isolates from patients with urological tumors were ESBL positive. Multivariate analysis showed that tumor metastasis (OR 3.41, 95%CI 1.84–6.30), urological cancer (OR 2.96, 95%CI 1.34–6.53), indwelling catheter (OR 2.08, 95%CI 1.22–3.55) and surgery or invasive manipulation (OR 1.98, 95%CI 1.13–3.50) were the independent risk factors. According to antimicrobial sensitivity, meropenem, imipenem and piperacillin/tazobactam were the most commonly used antibiotics for ESBL-producing *Enterobacteriaceae* UTI.

**Conclusions:**

In view of the high prevalence, clinicians should be alert to the occurrence of ESBL UTI, especially for patients with urological cancer or metastatic tumors. Regular replacement of urinary catheters, reduction of unnecessary invasive operations and selection of appropriate antibiotics are the necessary conditions to deal with the occurrence of ESBL UTI in adult cancer patients.

**Supplementary Information:**

The online version contains supplementary material available at 10.1186/s12879-023-08023-3.

## Introduction

Urinary tract infection (UTI) is one of the most common infectious diseases in the clinic [1]. Multi-drug resistance species producing extended-spectrum beta-lactamase (ESBL) is one of the most common pathogens causing nosocomial infection and obtained intensive clinical attention in recent years [2, 3].

ESBL-producing bacteria possess the following features: (1) inactivated penicillin and the third generation of cephalosporins antibiotics (e.g., ceftazidime and cefoperazone) by hydrolysis; (2) invalidated monocyclic β-lactam antibiotics (e.g., aztreonam and culumonen); (3) could not hydrolyze cephalomycin and carbapenems under normal circumstances; (4) could be suppressed by β-lactamase inhibitors (e.g., clavulanic acid, sulbactam, and tazobactam) [2, 4]. Generally, *Escherichia coli (E. coli)* and *Klebsiella pneumoniae* (*K. pneumoniae*) are two kinds of Gram-negative family *Enterobacteriaceae* with the highest frequency causing ESBL UTI [3, 5, 6]. Meanwhile, *Klebsiella oxytoca* (*K. oxytoca*) [7] and *Proteus mirabilis* (*P. mirabilis*) [1] are also included in the species of *Enterobacteriaceae* causing ESBL UTI.

Patients who suffered from cancer are a special group with the characteristics of immunological deficiency, high exhaustion, protracted course, and multiple complications [8]. The incidence rate of ESBL UTI involved cancer patients is continuing high [9, 10]. For a long time, the situation of ESBL related UTI referring patients with malignant tumors is of public concern on account that the failure of empirical treatments, which could cause multiple complications, interrupted therapeutic procedures, increase the economic burden, and prolonged illness duration [11]. Considering multiple procedures of radiotherapy and chemotherapy included in cancer therapy, the interruption by ESBL UTI may lead to distant metastasis of the tumor and loss of the opportunity for complete or partial remission ultimately.

Hence, it is critical to reasonably control UTI caused by ESBL**-**producing *Enterobacteriaceae* for tumor patients. For one thing, searching and identifying the associated risk factors of ESBL UTI is a convenient measure. For another, it is also essential to timely select and use appropriate antibiotics once ESBL UTI occurs in patients. Considering the above circumstances, we have sorted and processed the information for the specimens of urine culture positive in three cancer hospitals in Beijing from 2015 to 2019. In the current study, we describe the risk factors and predictors for ESBL-producing *Enterobacteriaceae* UTI related to cancer patients, as well as the clinical presentation, tumor characteristics, treatments, and antibiotic sensitivity over a 5-year period.

## Methods

### Study design

We conducted a retrospective, laboratory-based, multi-center study referring oncological patients suffering from UTI from January 2015 to December 2019 in Beijing, China. A total of three cancer hospitals participated in the study, including Cancer Hospital, Chinese Academy of Medical Sciences (CAMS), Beijing Chaoyang Sanhuan (SH) Cancer Hospital, and Cancer Hospital of Huanxing (HX) Chaoyang District Beijing. Cancer Hospital, CAMS served as a major tertiary-level referral center and clinic hospital for cancer patients in China. All three hospitals focused on the treatment of solid tumors and lymphoma, and none of them set up a hematological malignancy department other than lymphoma. All urine specimens were transported to the central laboratory (Department of Clinical Microbiology Laboratory, Cancer Hospital, CAMS) within two hours of collection for microbial culture. There are regular shuttle buses between the three hospitals, which can be driven in less than 10 min. The complete medical record was inquired from 2015 to 2019. The prevalence of ESBL UTI was observed for 5 years. Cancer patients older than 18 years who had underlying *E. coli*, *K. pneumoniae, K. oxytoca* or *P. mirabilis* related UTI were included in this study. Benign tumor cases, multi-bacteria UTI cases, episodes without antimicrobial susceptibility results, or complete case information were excluded from this study.

### Relevant definitions

UTI was defined as a positive urine culture (≥ 10^5^ colony-forming unit (CFU)/mL for clean-catch midstream urine or ≥ 10^4^ CFU/mL for catheterized urine) [6, 12]. Fever was defined as ≥ 37.3 ℃ of the axillary temperature. Invasive manipulation was identified according to a definition used previously: “requiring suture, incision, excision, or manipulation of tissues, of … usually, but not always… local, regional, or general anesthesia” [13, 14] such as intervention, puncture, intubation, drainage and so on. Chemotherapy referred to the use of chemotherapy agents during the treatment within half a year, such as intravenous infusion, abdominal infusion, bladder infusion and so on.

### Data collection

A new medical records information system had been launched since 2015. Therefore, the complete medical records information for multivariate analysis was acquired from 2015. The hospital medical records retrieval system was used to query the corresponding clinical data of all patients, including age, gender, tumor pathological type, clinical stage, underlying diseases, treatment methods, temperature, and prognosis, etc. The laboratory information system was used to acquire the various laboratory test indicators and antimicrobial susceptibility results.

### Species identification, antimicrobial susceptibility testing and ESBL confirmation testing

Species identification and antimicrobial susceptibility testing were performed using NMIC/ID4 card with BD Phoenix^TM^100 Automated Microbiology System (Becton, Dickinson and Company, Sparks, Maryland, USA). The following antimicrobial agents were tested: amikacin (8 ~ 32 μg/mL), amoxicillin/clavulanate (4/2 ~ 16/8 μg/mL), ampicillin (4 ~ 16 μg/mL), ampicillin/sulbactam (4/2 ~ 16/8 μg/mL), aztreonam (2 ~ 16 μg/mL), cefepime (2 ~ 16 μg/mL), cefotaxime (1 ~ 32 μg/mL), ceftazidime (1 ~ 16 μg/mL), ciprofloxacin (0.5 ~ 2 μg/mL), gentamicin (2 ~ 8 μg/mL), imipenem (1 ~ 8 μg/mL), levofloxacin (1 ~ 8 μg/mL), meropenem (1 ~ 8 μg/mL), piperacillin/tazobactam (4/4 ~ 64/4 μg/mL), tetracycline (2 ~ 8 μg/mL), trimethoprim/sulfamethoxazole (0.5/9.5 ~ 2/38 μg/mL), cefotaxime/clavulanate (for ESBL, < 9 μg/mL), ceftazidime/ clavulanate (for ESBL, < 9 μg/mL), cefpodoxime-proxetil (for ESBL, < 9 μg/mL), ceftazidime (for ESBL, < 9 μg/mL) and ceftriaxone/clavulanate (for ESBL, < 9 μg/mL) [15]. If the MIC of ciprofloxacin was less than or equal to 0.5 μg/mL, or the MIC of levofloxacin was less than or equal to 1 μg/mL, sensitivity or intermediation was confirmed by disk diffusion test (ciprofloxacin (5 μg) and levofloxacin (5 μg), respectively). Phenotypic ESBL confirmation was performed with a double-disk synergy test (cefotaxime (30 μg), cefotaxime/clavulanic acid (30 μg/10 μg), ceftazidime (30 μg) and ceftazidime/clavulanic acid (30 μg/10 μg) disk) following clinical and laboratory standards institute (CLSI) criteria [16]. The recently revised CLSI species-specific clinical breakpoints (CBPs) were used to interpret the MIC results. The quality control strains were *Escherichia coli* ATCC25922, *Pseudomonas aeruginosa* ATCC27853 and *Klebsiella pneumoniae* ATCC700603.

### Statistical analysis

Continuous variables were evaluated with the student’s *t* test or Mann–Whitney *U* test for independent samples, and categorical variables were compared with the chi-square test or Fisher’s exact test. Associations that were found to be significant enough *P* < 0.05 in univariate analysis were further analyzed by multivariate logistic regression to identify independent risk factors. Statistical significance was regulated as 2-tailed *P* < 0.05. All the data were analyzed with version 22.0 of IBM SPSS statistics.

## Results

### Study participants

**Fig. 1:**
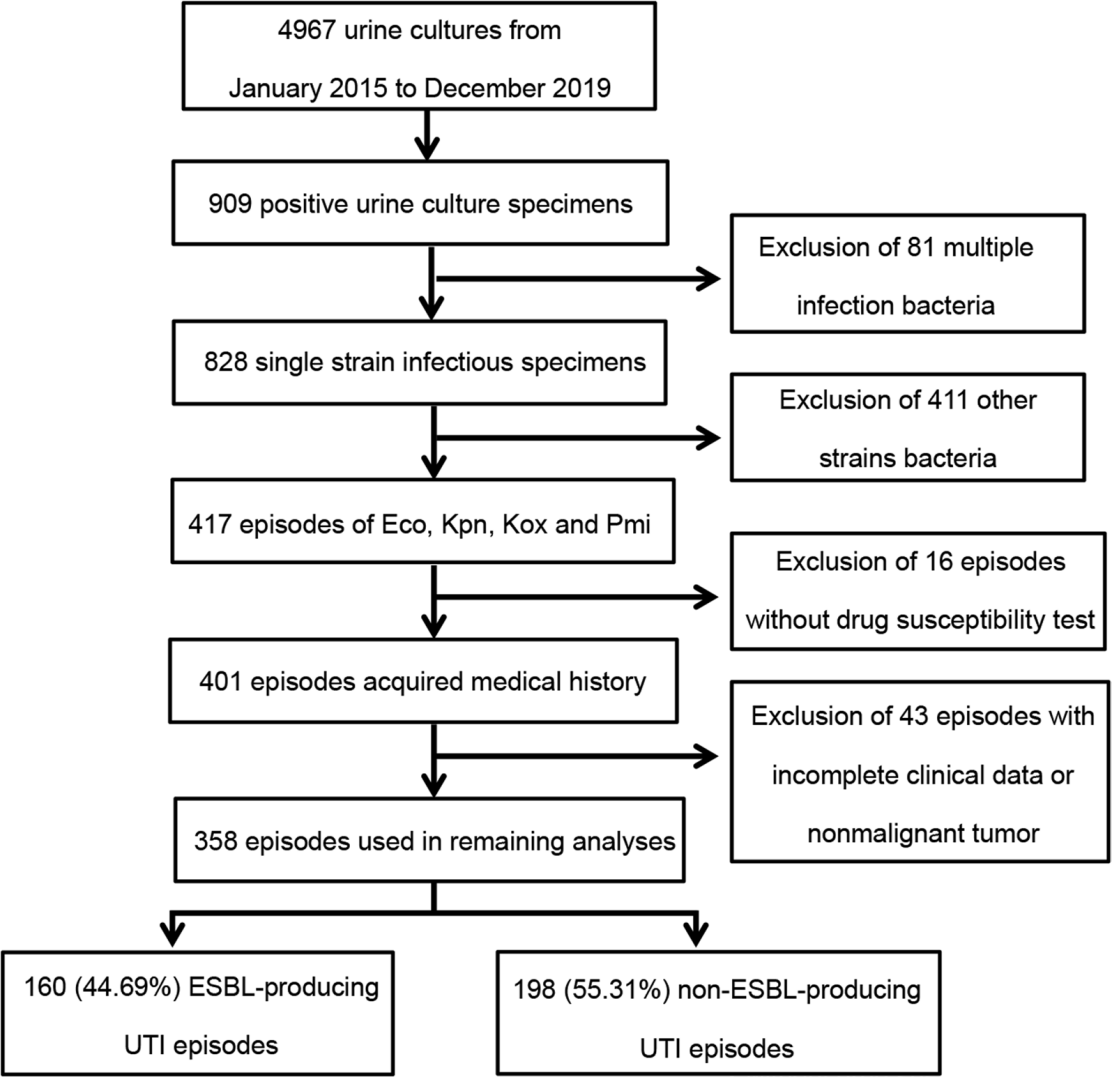
Flowchart of patients inclusion in this study. *Eco* Escherichia coli*, Kpn* Klebsiella pneumoniae*, Kox* Klebsiella oxytoca, *Pmi* Proteus mirabilia, *ESBL* extended-spectrum beta lactamase, *UTI* urinary tract infection

A total of 4967 specimens were received and performed urine culture tests from 1 January 2015 to 31 December 2019 demonstrated in Fig. 1. Out of these, 909 (18.30%) were positive for urine culture. After 81 episodes of multiple-bacterial infection were excluded, 828 episodes of single-bacterial infection were further analyzed. Among the 828 episodes, 417 (50.36%) episodes belonged to the range of *E. coli*, *K. pneumoniae*, *K. oxytoca* and *P. mirabilis*. Further, after excluding 16 episodes without antimicrobial susceptibility testing results, and 43 episodes with incomplete medical records or proved to be nonmalignant tumor patients, 358 episodes met the inclusion criteria. Ultimately, 160 episodes were included in the ESBL group while 198 were in the non-ESBL group.

### Change of ESBL prevalence state

**Fig. 2:**
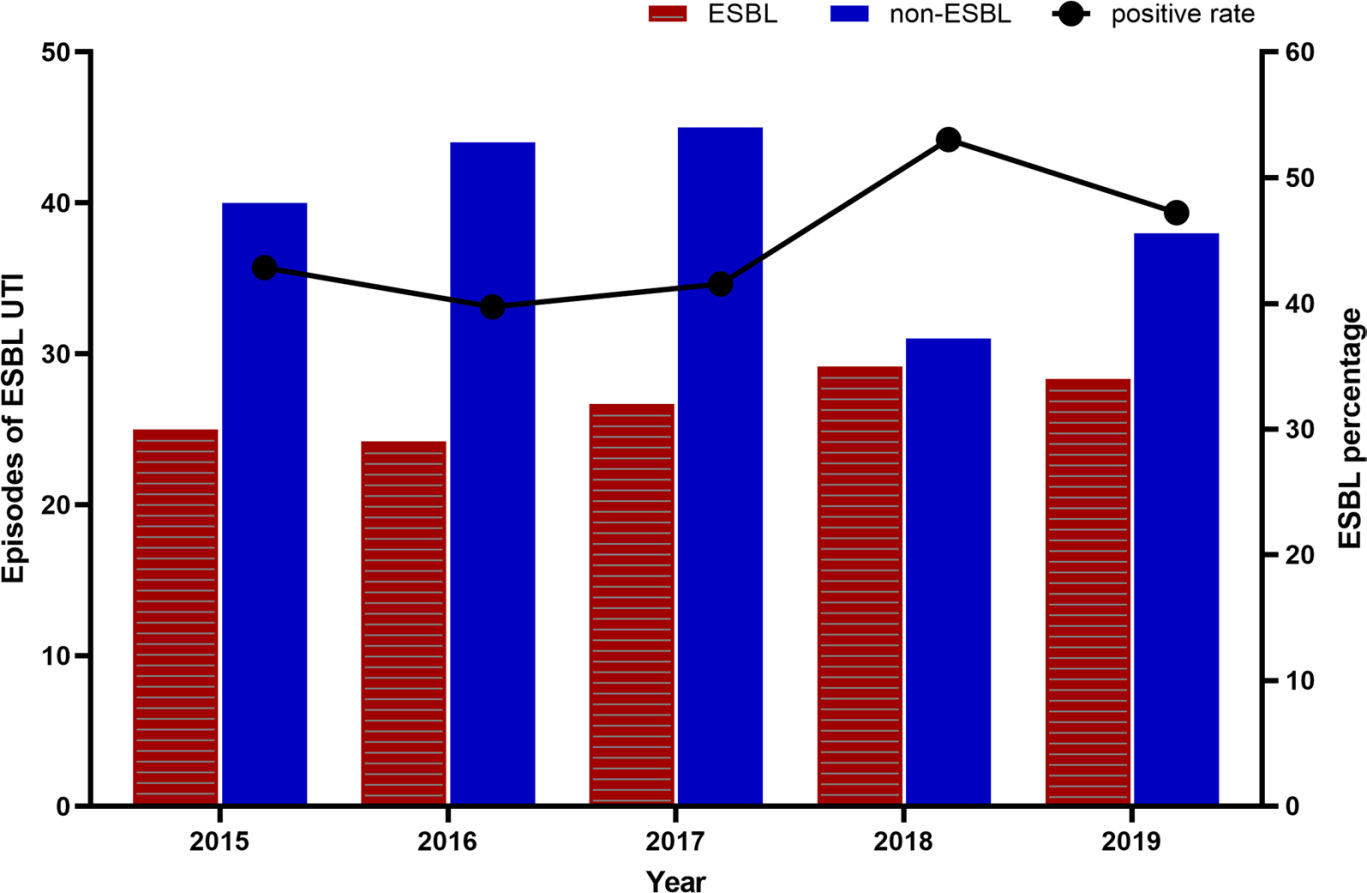
The prevalence of ESBL-caused UTIs each year. *ESBL* extended-spectrum beta lactamase, *UTI* urinary tract infection

The prevalence and proportion of ESBL and non-ESBL UTI for each year from 2015 to 2019 were demonstrated in Fig. 2. During the five years, the peak of the episodes of ESBL UTI was present in 2018. While the highest number of non-ESBL appeared in the year 2017. Roughly, the ESBL prevalence fluctuated between 40 and 53%. The peak of the positive rate for ESBL UTI occurred in 2018 (53.03%), while the bottom was in 2016 (39.73%).

Considering the composition of pathogenic bacteria in 358 episodes, *E. coli* was the highest percentage whether in ESBL group (82.50%) or non-ESBL group (73.23%). *K. pneumoniae* ranked the second place, accounting for 16.82% in ESBL group and 17.17% in non-ESBL group. In addition, only one episode (0.63%) was *K. oxytoca* in ESBL group, while 15 episodes (7.58%) were *P. mirabilis* and 4 episodes (2.02%) were *K. oxytoca* in non-ESBL group (Additional file 1: Fig. S1).

The prevalence and distribution of different species for ESBL-producing bacteria for 5 years were further analyzed (Table 1). From 2015 to 2019, the positive rate of *E.coli* in ESBL group fluctuated between 40.98 (2017) and 54.00% (2015). For ESBL-producing *K. pneumoniae,* the highest positive rate (63.64%) appeared in 2017, while the lowest positive rate (23.08%) occurred in 2015. *P. mirabilis* and *K. oxytoca* were basically appeared in non-ESBL group.

**Table 1:** The prevalence and distribution of four kinds of species for ESBL and non-ESBL UTI

Year	*Eco*			*Kpn*			*Kox*			*Pmi*		
	Total	ESBL	PR (%)	Total	ESBL	PR (%)	Total	ESBL	PR (%)	Total	ESBL	PR (%)
2015	50	27	54.00	13	3	23.08	1	0	0	6	0	0
2016	51	23	45.10	17	6	35.29	1	0	0	4	0	0
2017	61	25	40.98	11	7	63.64	1	0	0	4	0	0
2018	56	30	53.57	7	4	57.14	2	1	50.00	1	0	0
2019	59	27	45.76	13	7	53.85	0	0	0	0	0	0

*Eco* Escherichia coli*, Kpn* Klebsiella pneumoniae*, Kox* Klebsiella oxytoca, *Pmi* Proteus mirabilia, *ESBL* extended-spectrum beta-lactamase, *UTI* urinary tract infection, *PR* positive rate of ESBL

### Demographic characteristics

The average age in the ESBL group were 58.53 ± 13.59 while 59.35 ± 11.21 year in the non-ESBL group. Age composition did not differ significantly in the two groups (Table 2). Similarly, there was no statistically significant difference in the two groups referring to gender composition (*P* = 0.098) and hospital area (*P* = 0.805), respectively. Due to the particularity of multi-cycle therapy for malignant tumor patients, such as radiotherapy and chemotherapy, almost all patients were hospitalized. The univariate analysis showed that surgery or invasive manipulation within 6 months (*P* = 0.012), urethral catheterization (*P* < 0.001), leukocytes increased (*P* = 0.002) and protein-positive (*P* = 0.006) in routine urinalysis were significantly higher in the ESBL group, but erythrocytes increased (*P* < 0.001) was more frequent in the non-ESBL group.

**Table 2:** Epidemiological and clinical characteristics compared of ESBL and non-ESBL UTI in cancer patients

Description	Total (n = 358)	ESBL (n = 160) n(%)	Non-ESBL (n = 198) n (%)	*P*-value
Age, years	58.98 ± 12.32	58.53 ± 13.59	59.35 ± 11.21	0.536
Gender, Male	144 (40.2)	72 (45.0)	72 (36.4)	0.098
Hospital areas				0.805
CAMS	94 (26.3)	42 (26.3)	52 (26.3)	
SH	77 (21.5)	32 (20.0)	45 (22.7)	
HX	187 (52.2)	86 (53.8)	101 (51.0)	
Smoking	95 (26.5)	44 (27.5)	51 (25.8)	0.710
Diabetes	74 (20.7)	35 (21.9)	39 (19.7)	0.613
Fever (≥ 37.3℃)	87 (24.3)	44 (27.5)	43 (21.7)	0.205
Chemotherapy within 6 months	234 (65.4)	93 (58.1)	120 (60.6)	0.634
Surgery or invasive manipulation within 6 months	129 (36.0)	69 (43.1)	60 (30.3)	0.012
Radiotherapy within 6 months	77 (21.5)	31 (19.4)	46 (23.2)	0.377
Received antibiotics within 1 week	192 (53.6)	90 (56.3)	102 (51.5)	0.372
Urethral catheterization	194 (54.2)	106 (66.3)	88 (44.4)	< 0.001
Used glucocorticoids within 2 weeks	161 (45.0)	68 (42.5)	93 (47.0)	0.398
Hematologic analysis				
Leukocyte reduced	23 (6.4)	10 (6.3)	13 (6.6)	0.904
Hemoglobin reduced	173 (48.3)	82 (51.3)	91 (46.0)	0.319
Serum creatinine increased	10 (2.8)	3 (1.9)	7 (3.5)	0.522
Plasma albumin reduced	114 (31.8)	52 (32.5)	62 (31.3)	0.811
Routine urinalysis^a^				
Leukocytes increased	245 (71.4)	120 (80.0)	125 (64.8)	0.002
Protein positive	166 (48.4)	85 (56.7)	81 (42.0)	0.006
Erythrocytes increased	226 (65.9)	81 (54.0)	145 (75.1)	< 0.001
Bacteriuria increased	268 (78.1)	122 (81.3)	146 (75.6)	0.098

*ESBL* extended-spectrum beta lactamase, *UTI* urinary tract infection, *CAMS* Cancer hospital, Chinese academy of medical science, *SH* Beijing Chaoyang Sanhuan Cancer Hospital, *HX* Cancer Hospital of Huanxing Chaoyang District Beijing

^a^343 episodes had the detection results of routine urinalysis, including 150 in ESBL group and 193 in non-ESBL group

Leukocyte reduced < 2.0 × 10^9^/L white blood cells in routine blood test, Hemoglobin reduced < 110 g/L hemoglobin in routine blood test, Serum creatinine increased > 133 umol/L creatinine in serum, Plasma albumin reduced ≤ 35.0 g/L plasma albumin, Leukocytes increased ≥ 10 leukocytes/HP in microscopy by sediment urinalysis, Protein positive ≥ 1 + by Dry Chemistry, Erythrocytes increased ≥ 10 erythrocytes/HP in microscopy by sediment urinalysis, Bacteriuria increased > 340 /uL by formed elements urinalysis

### Tumor clinical characters

For the three hospitals participating in this study, there were no patients with hematological malignancies other than lymphoma. Among the 358 episodes, 334 (93.3%) suffered solid tumors while 24 (6.7%) had lymphoma (Table 3). The tumor types (*P* = 0.001) and clinical stages (*P* = 0.028) differed significantly between ESBL group and non-ESBL group. Subgroup analysis by tumor type revealed that 62.5% of isolates from patients with urological tumors were ESBL positive. Among them, prostate cancer and bladder cancer were the two tumor types most associated with ESBL positivity. The ESBL-positive rates of isolates from prostate and bladder cancer patients were 77.8% and 64.5%, respectively. For pathological type, patients with adenomatous carcinoma and squamous cell carcinoma account for the largest proportion in both ESBL (69.4%) and non-ESBL (69.7%) groups. The proportion of patients with clinical stage IV, nearly half, was similar between the two groups.

**Table 3:** Comparison of tumor clinical characters between ESBL and non-ESBL among 358 UTI episodes

Tumor information	Total, n	ESBL n (%)	Non-ESBL n (%)	*P*-value
*Tumor types*	358	160 (44.7)	198 (55.3)	0.001
Urological cancer	64	40 (62.5)	24 (37.5)	0.002
Prostate cancer	18	14 (77.8)	4 (22.2)	
Bladder cancer	31	20 (64.5)	11 (35.5)	
Ureter and renal pelvis cancer	4	2 (50.0)	2 (50.0)	
Penile cancer	2	1 (50.0)	1 (50.0)	
Renal cancer	9	3 (33.3)	6 (66.7)	
Gynecological cancer	73	37 (50.7)	36 (49.3)	0.248
Cervical caner	54	29 (53.7)	25 (46.3)	
Ovarian and ureter cancer	14	7 (50.0)	7 (50.0)	
Endometrial cancer	5	1 (20.0)	4 (80.0)	
Gastrointestinal neoplasms	85	38 (44.7)	47 (55.3)	0.998
Rectal cancer	33	18 (54.5)	15 (45.5)	
Colorectal cancer	19	9 (47.4)	10 (52.6)	
Esophagus cancer	9	3 (33.3)	6 (66.7)	
Stomach cancer	9	3 (33.3)	6 (66.7)	
Liver cancer	6	2 (33.3)	4 (66.7)	
Pancreatic cancer	6	2 (33.3)	4 (66.7)	
Gallbladder cancer	3	1 (33.3)	2 (66.7)	
Hematologic malignancy	24	8 (33.3)	16 (66.7)	0.247
Lymphoma	24	8 (33.3)	16 (66.7)	
Thoracic neoplasms	89	25 (28.1)	64 (71.9)	< 0.001
Breast cancer	19	7 (36.8)	12 (63.2)	
Lung cancer	70	18 (25.7)	52 (74.3)	
Other	23	12 (52.2)	11 (47.8)	0.456
Pathological type	358	160	198	0.137
Adenomatous carcinoma	175	71 (40.6)	104 (59.4)	
Squamous cell carcinoma	74	40 (54.1)	34 (45.9)	
Neuroendocrine carcinoma	31	11 (35.5)	20 (64.5)	
Urothelial carcinoma	31	19 (61.3)	12 (38.7)	
Non-Hodgkin lymphoma	24	8 (33.3)	16 (66.7)	
Sarcoma	8	4 (50.0)	4 (50.0)	
Other	15	7 (46.7)	8 (53.3)	
Clinical stages	358	160	198	0.028
I	24	10 (41.7)	14 (58.3)	
II	32	11 (34.4)	21 (65.6)	
III	79	26 (32.9)	53 (67.1)	
IV	223	113 (50.7)	110 (49.3)	

### Risk factors for ESBL-producing UTI in cancer patients

The multi-variable logistic regression analysis was conducted, and the predictors included patient profiles (age, sex, different hospital areas, the temperature exceeded 37.3 Celsius, smoke, used glucocorticoids, suffered diabetes), tumor-related information (metastasis, pathological types, tumor types), treatment (surgical or invasive manipulation in the preceding 6 months, radiotherapy or chemotherapy in the preceding six months, urethral catheterization, received antibiotics within one week), as well as several detected results in blood and urine routine tests. After analysis, the results indicated that tumor metastasis (OR 3.41, 95%CI 1.84–6.30), urological cancer (OR 2.96, 95%CI 1.34–6.53), indwelling catheter (OR 2.08, 95%CI 1.22–3.55) and surgery or invasive manipulation within half a year (OR 1.98, 95%CI 1.13–3.50) were the independent risk factors of ESBL**-**producing *Enterobacteriaceae* UTI for cancer patients (Table 4).

**Table 4:** Risk factors of ESBL UTI determined by multivariable logistic regression

Variable	Crude Odds ratio (95% CI)	Adjusted Odds ratio (95% CI)	*P* values
Different sex	0.698 (0.456 1.069)	1.007 (0.478 2.121)	0.986
Age ≥ 60 years old	0.980 (0.646 1.487)	0.794 (0.455 1.385)	0.416
Different hospital areas (ref. CAMS)			0.960
SH	0.880 (0.479 1.619)	0.889 (0.392 2.015)	0.779
HX	1.054 (0.641 1.735)	0.938 (0.489 1.798)	0.847
Temperature ≥ 37.3 Celsius	1.367 (0.842 2.219)	1.034 (0.574 1.862)	0.912
Addicted to smoking	1.093 (0.683 1.751)	0.789 (0.363 1.718)	0.551
Used glucocorticoids within 2 weeks	0.835 (0.548 1.270)	0.785 (0.466 1.323)	0.364
Suffered from diabetes	1.142 (0.683 1.907)	1.513 (0.803 2.848)	0.200
Metastasis (clinical stage IV)	1.923 (1.238 2.989)	3.406 (1.841 6.303)	** < 0.001**
Pathological types **(**ref. others**)**			0.231
Adenomatous carcinoma	0.719 (0.420 1.229)	0.939 (0.484 1.824)	0.853
Squamous cell carcinoma	1.238 (0.655 2.342)	2.305 (0.878 6.047)	0.090
Neuroendocrine carcinoma	0.579 (0.245 1.367)	1.280 (0.424 3.867)	0.661
Tumor types** (**ref. others**)**			0.026
Urological cancer	2.771 (1.560 4.923)	2.958 (1.339 6.533)	**0.007**
Gynecological cancer	1.709 (1.002 2.913)	1.018 (0.441 2.346)	0.967
Surgical therapy or invasive manipulation within half a year	1.744 (1.128 2.696)	1.984 (1.126 3.495)	**0.018**
Radioactive therapy within half a year	0.794 (0.476 1.325)	0.901 (0.443 1.833)	0.774
Chemotherapy within half a year	0.902 (0.590 1.379)	1.037(0.580 1.852)	0.903
Indwelling catheter	2.454 (1.594 3.777)	2.081 (1.222 3.546)	**0.007**
Received antibiotics within 1 week	1.210 (0.796 1.839)	1.211 (0.715 2.053)	0.476
Hematologic analysis			
Detected leukocyte reduced	0.949 (0.405 2.224)	1.006 (0.383 2.645)	0.990
Detected hemoglobin reduced	1.236 (0.814 1.876)	1.352 (0.794 2.303)	0.266
Plasma albumin reduced	1.056 (0.676 1.651)	0.659 (0.370 1.173)	0.156
Serum creatinine increased	0.521 (0.133 2.050)	0.395 (0.089 1. 766)	0.224
Routine urinalysis			
Detected leukocytes increased	2.176 (1.323 3.578)	1.560 (0.812 3.000)	0.182
Detected protein positive	1.808 (1.175 2.783)	0.833 (0.456 1.521)	0.553
Detected erythrocytes increased	2.573 (1.628 4.068)	1.744 (0.952 3.196)	0.072
Detected bacteriuria increased	1.403 (0.829 2.374)	0.916 (0.457 1.835)	0.805

*CI* confidence interval, *ref* reference group

### Antibiotic susceptibility

**Fig. 3:**
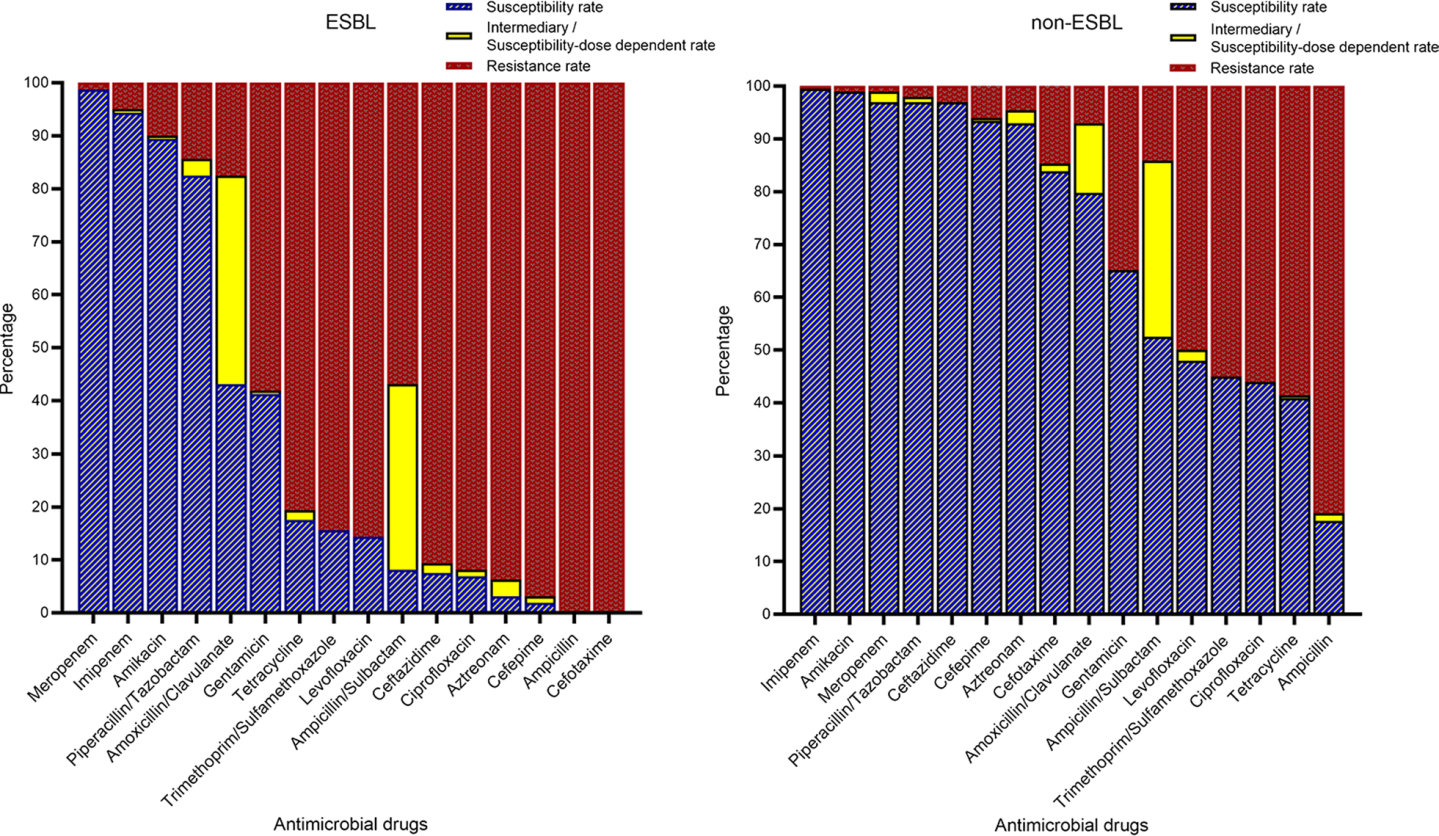
Antibiotic susceptibility of bacteria isolates stratified by ESBL and non-ESBL UTIs in cancer patients. *ESBL* extended-spectrum beta lactamase, *UTI* urinary tract infection

The differences in susceptibility to commonly used antibiotics between the ESBL and the non-ESBL groups were shown in Fig. 3. All ESBL isolates showed uniformly resistance to ampicillin and cefotaxime, as well as 96.88% resistance to cefepime. For fluoroquinolones, the resistance rates of ESBL group to levofloxacin and ciprofloxacin were 85.62% and 91.88%, respectively. Generally, compared with non-ESBL, ESBL-positive isolates were observably more resistant to the overwhelming majority of antibiotics in this study. Nevertheless, almost all ESBL-positive isolates were proved susceptible to meropenem (98.75%) and imipenem (94.38%). For piperacillin/tazobactam, the sensitivity rate of the ESBL group was 82.50%.

## Discussions

Compared with the ESBL-related bloodstream infection (BSI) [17, 18], the research for ESBL UTI in cancer patients was finite. A large percentage of studies for ESBL UTI was focused on children [6, 19, 20] or adults in community [21, 22]. The analysis of ESBL UTI involving tumors was partially hematological malignancy [23, 24], with few studies on solid tumors. However, it should not be ignored of the ESBL**-**producing *Enterobacteriaceae* UTI in solid tumor sufferers. On one hand, once cancer patients developed ESBL UTI, the anti-tumor treatment could be delayed, suspended, or even failed. On the other hand, the immunity of tumor patients was low, and some in cachexia, which could cause UTI persistent, repeatedly infected, and even bring about systemic infection and death. Therefore, it is necessary to identify the associated risk factors for ESBL UTI in cancer patients.

Although the mortality of ESBL UTI was not as serious as ESBL BSI, the incidence was consistently high [25, 26]. During the five years, the prevalence of ESBL-producing *Enterobacteriaceae* UTI in cancer patients was stable basically, circled around 40 to 53% roughly (Fig. 2). The lowest one occurred in 2016 with a positive rate of 39.73%. From 2015 to 2019, the average probability of ESBL infection was 44.69%, which was a high percentage but similar to the results in several studies about non-tumor patients. [19, 20, 27]. As far as oncological patients were concerned, the related report of the prevalence of ESBL UTI was indeed rare. The scanty study about pediatric oncology with an incidence approaching of 40% [24], which seems to be near to the years with low prevalence in our study.

Generally, *E. coli* occupied the absolute predominance (82.50%) in ESBL group (Additional file 1: Fig. S1), which served as the main contribution to the overall trend in the prevalence of ESBL UTI in our study. *K. pneumoniae,* with a ratio of 16.82%, took subsidiary effects on the overall prevalence of ESBL UTI for five years. Considering the distribution of different species (Table 1), the proportion and quantity of *E. coli* were basically stable for each year ranging from 2015 to 2019. However, the proportion of ESBL-related *K. pneumoniae* was changed obviously of its limited number. The slight change in the number of *K. pneumoniae* could affect its positive rate visibly.

In view of the particularity of the crowd of cancer patients, the basic information and detection results were collected (Table 2). Meanwhile, the tumor-elated specific condition was gathered (Table 3). The clinical types classified by the system and the pathological types grouped by histopathology were demonstrated in Table 3. For the convenience of subsequent multivariate analysis (Table 4), we divided multifarious tumor types into three groups including gynecological tumors, urological tumors and others. Neoplasm stages of I–III were merged into the pre-metastatic group, while the IV period was brought into the metastasis group in order to facilitate subsequent analysis. Involved in pathologic types, adenomatous carcinoma, squamous cancer and neuroendocrine carcinoma were the three most common types. The remaining types were classified into other groups.

Multivariate analysis revealed that there were significant differences referring to metastasis, tumor types, urethral catheterization and surgery or invasive operation between ESBL and non-ESBL groups. The independent risk factor most associated with ESBL UTI was tumor metastasis (OR 3.41, 95%CI 1.84–6.30), which had been reported previously [28]. Several reasons may be involved. For one thing, the probability of UTI increased greatly because of a long therapeutic procedure and multiple treatments of stage IV cancer patients [29]. A variety of antitumor drugs have been reported to have antibacterial activity in the research stage [30, 31], so the use of certain antitumor drugs may induce bacteria to develop antimicrobial resistance. For another, when the tumor develops into Phase IV, the patients were in a state of high consumption and poor nutrition. The ability of the immune system to resist the invasion of external pathogens was strongly reduced, which served as convenient conditions for ESBL-related flora colonization [32]. Additionally, tumor metastasis was accompanied by the change of micro-ecological environment in patients [33], which was also the reason why ESBL UTI was easy to occur.

Another independent risk factor closely associated with ESBL UTI was urological cancer (OR 2.96, 95%CI 1.34–6.53). It was not difficult to perceive, the incidence of UTI for urinary system tumors was higher than in other systems due to the characteristic of anatomical proximity. No matter direct invasion or indirect oppression, urological neoplasms could cause urine retention, increasing the probability of bacterial infection [34, 35]. Additionally, long-term repeated infection, the change of urinary flora and the destruction of the inherent barrier are all the reasons why ESBL UTI is higher in urological tumors than others [36].

Undoubtedly, indwelling catheter (OR 2.08, 95%CI 1.22–3.55) was also observed to be an independent high-risk factor for ESBL UTI in cancer patients. This has been confirmed by many previous studies [25, 35, 37]. ESBL-producing *E. coli* colonized on the skin of the urethral orifice during long-term indwelling of the urinary catheter may damage the urethral mucosa and lead to UTI [36]. It suggested that the indications of indwelling urinary catheter should be strictly controlled in clinical work. Patients with indwelling urinary catheters should replace the urinary catheter regularly to minimize the damage to urethral mucosa caused by catheter removal or re-intubation. Surgery and invasive procedures (OR 1.98, 95%CI 1.13–3.50) had an impact on the occurrence of ESBL UTI, which was consistent with previous reports [38, 39]. The above operation process was easy to break the immune barrier of patients and increase the risk of UTI.

From the results of antimicrobial susceptibility (Fig. 3), the antimicrobial resistance of ESBL-positive isolates to cephalosporins was almost 100%. In addition, the resistance rate of ESBL-positive isolates to levofloxacin, a commonly used antibiotic for the treatment of urinary tract infections, was higher than 85%. The more sensitive antibiotics were aminoglycosides (amikacin), carbapenems (imipenem, meropenem) and sulfonamides (cotrimoxazole). However, carbapenems were still the first choice in clinical treatment. Because of the severe nephrotoxicity (inducing drug-induced renal failure) of aminoglycosides [40], the application of it was limited, especially for tumor patients. Although the sensitivity rate of it was higher than others, the probability of clinical choices was relatively low. They were rarely used alone, instead of combining them with other antibiotics. Meanwhile, the adverse reaction of sulfonamides was a prominent problem, which led to its obvious limitations in clinical application. Adverse reactions were mainly manifested in urinary system damage, allergic reaction, blood system and nervous system reaction, liver damage and even acute liver necrosis [40]. Due to the great side effects on the liver and kidney function of sulfonamides, cancer patients, with more basic diseases and weaker immunity, rarely chose them in clinical treatment. The antibiotics used for the first time were mainly cephalosporins for non-ESBL UTI patients and the symptoms improved during the treatment. Among the patients with ESBL UTI who had improved or cured after treatment, meropenem, imipenem and piperacillin/tazobactam were both the most commonly used antibiotics. The three antibiotics could be used as empirical drugs for the clinical treatment of ESBL UTI.

This study included the following limitations. First, due to the retrospective study, incomplete clinical data or medical records could not be supplemented. The use of antibiotics outside the hospital was unavailable because the multiple treatment intervals for cancer patients were often out of the hospital. Second, the strains involved in the study were not preserved. Therefore, the prevalent sequence types of the ESBL-producing *Enterobacteriaceae* strains causing UTI in cancer patients could not be detected and analyzed. Third, various chemotherapeutic drugs were involved in this study. Therefore, it was complicated to analyze the relationship between the types of chemotherapeutic drugs and ESBL UTI.

## Conclusion

In conclusion, considering the high prevalence of ESBL**-**producing *Enterobacteriaceae* UTI over 40% of cancer patients, it was necessary to seek out the relevant risk factors. After multivariate analysis, metastasis, urological tumors, catheterization and surgery or invasive procedures were considered to be the risk factors of ESBL**-**producing *Enterobacteriaceae* UTI. Therefore, once UTI appears in patients with metastatic urological tumors, it should be alert to the occurrence of ESBL-related infection. At the same time, the use of urinary catheters and invasive operations should be reduced as much as possible.

## Supplementary Information

**Additional file 1: Figure S1.** The composition of pathogenic bacteria for ESBL and non-ESBL UTIs in cancer patients.

## Data Availability

Datasets used and/or analysed during the current study are available from the corresponding author on reasonable request.
