# Supplementary material for: Extended-spectrum beta-lactamase-producing Enterobacteriaceae related urinary tract infection in adult cancer patients: a multicenter retrospective study, 2015–2019
Source: BMC Infect Dis. 2023 Mar 6;23:129. doi: 10.1186/s12879-023-08023-3 (PMC9987039; doi:10.1186/s12879-023-08023-3)
Supplement: Supplementary file 1 — Additional file 1: Figure S1. The composition of pathogenic bacteria for ESBL and non-ESBL UTIs in cancer patients. [file 12879_2023_8023_MOESM1_ESM.docx]

**Fig** **S1:** The composition of pathogenic bacteria for ESBL and non-ESBL UTIs in cancer patients

*Eco* Escherichia coli*, Kpn* Klebsiella pneumoniae*, Kox* Klebsiella oxytoca, *Pmi* Proteus mirabili

*ESBL* extended-spectrum beta lactamase-producing, *UTI* urinary tract infection

**
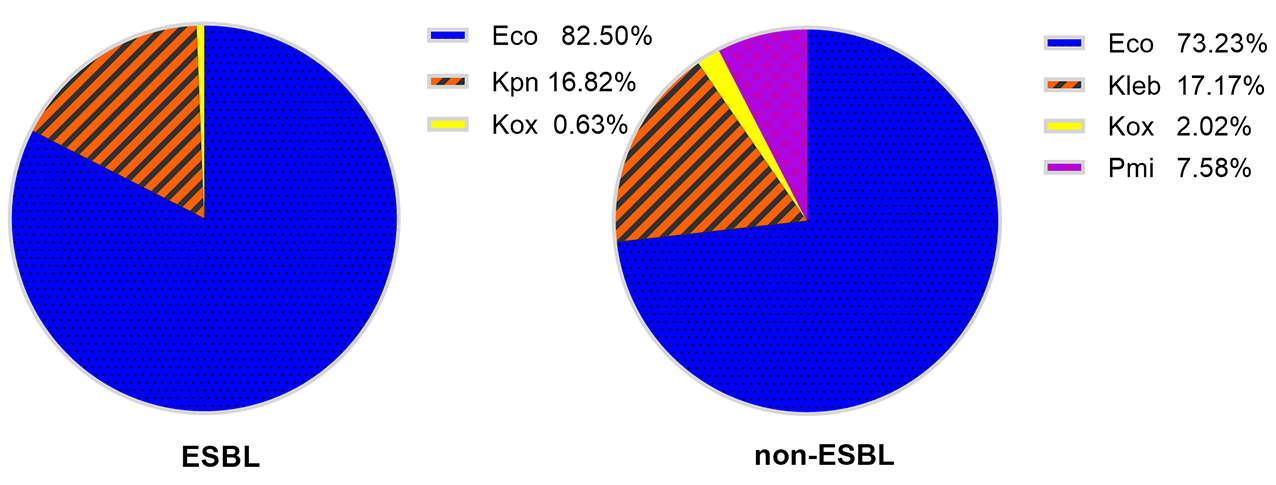
**
